# The duality of veterinary career satisfaction: shared domains of fulfillment and distress in veterinary practice

**DOI:** 10.3389/fvets.2026.1958614

**Published:** 2026-10-08

**Authors:** Sara Ritzie, Andria Jones, Lauren E. Van Patter, Jason Coe, Elizabeth C. Kurucz, Lynn Henderson, Shane Bateman

**Affiliations:** 1 Department of Clinical Studies, Ontario Veterinary College, University of Guelph, Guelph, ON, Canada; 2 Department of Population Medicine, Ontario Veterinary College, University of Guelph, Guelph, ON, Canada; 3 Gordon S. Lang School of Business and Economics, University of Guelph, Guelph, ON, Canada

**Keywords:** career satisfaction, moral distress, professional culture, professional fulfillment, qualitative research, spectrum of care, veterinary wellbeing

## Abstract

**Introduction:**

Veterinary medicine is associated with both substantial professional rewards and significant occupational challenges. While previous research has identified contributors to professional satisfaction and distress, these factors are commonly discussed as distinct positive or negative influences. During a broader study evaluating a spectrum of care (SpOC) workshop, an unexpected pattern emerged regarding how participants experienced career satisfaction.

**Methods:**

This secondary qualitative analysis examined semi-structured interviews and questionnaire open-text responses collected during a mixed-methods study exploring implementation of SpOC in veterinary practice. Data were analyzed using an inductive reflexive thematic analysis approach.

**Results:**

Participants did not describe separate sources of satisfaction and dissatisfaction. Instead, the same domains of veterinary practice were experienced as either fulfilling or distressing depending on context. Supportive team relationships, professional autonomy, value alignment, and flexibility in care planning appeared to shift experiences toward satisfaction, whereas judgment, organizational constraints, fear of professional repercussions, and misalignment between values and achievable actions contributed to distress. Participants frequently described SpOC as helping reconcile competing obligations to patients, clients, colleagues, employers, and themselves, thereby reducing ethical tension and supporting value-aligned practice. However, fear and elements of the hidden curriculum emerged as important barriers limiting the adoption of flexible approaches to care.

**Discussion:**

These findings suggest that veterinary wellbeing may be better understood as a dynamic, context-dependent phenomenon in which fulfillment and distress arise from shared domains of professional practice rather than distinct positive and negative workplace factors. By supporting value-aligned decision-making within real-world constraints, SpOC may represent one mechanism through which practitioner wellbeing and access to care can be advanced simultaneously.

## 1 Introduction

Veterinary medicine is a profession characterized by both profound rewards and significant challenges. Veterinarians commonly report a strong sense of purpose, intellectual engagement, and satisfaction derived from helping animals and supporting clients (1–6), while simultaneously experiencing elevated rates of burnout, compassion fatigue, moral distress, and other adverse mental health outcomes (4, 5, 7–13). Previous research has identified a wide range of factors associated with wellbeing, including client relationships, workplace culture, ethical conflict, clinical outcomes, and opportunities for professional growth. These factors are often discussed as either contributors to satisfaction or contributors to distress.

Considerable research has focused on identifying risk factors for poor mental health and protective factors that promote resilience and job satisfaction. Studies examining compassion satisfaction, burnout, and moral distress have provided important insights into the challenges and rewards associated with veterinary work (1–6, 8–13). However, much of this literature implicitly treats sources of satisfaction and distress as distinct constructs. Less attention has been paid to whether professional satisfaction and distress arise from distinct domains of practice or whether they represent different manifestations of the same aspects of veterinary work. Understanding this distinction may be important for designing interventions that promote wellbeing because it shifts attention from identifying inherently positive or negative aspects of veterinary work towards understanding the nuanced conditions under which common experiences become fulfilling or distressing.

Several findings within the existing literature suggest this relationship may be more complex than a simple separation of positive and negative workplace influences. The concept of compassion satisfaction recognizes that caregiving relationships can generate meaning, resilience, and professional fulfillment (1–4), while compassion fatigue literature highlights how those same relationships may contribute to emotional exhaustion and distress (8, 14–17). Similarly, studies exploring meaning in veterinary work have identified client relationships, professional competence, intellectual challenge, and contribution to animal welfare as important sources of wellbeing (1, 4, 9, 18), while parallel bodies of literature describe these same domains as contributors to stress, burnout, and moral conflict (19–21). This apparent paradox suggests that many of the experiences shaping wellbeing are neither inherently positive nor negative. Instead, the impact of common professional demands may depend on how they are experienced within specific organizational, interpersonal, and cultural contexts. Although previous studies have documented both rewarding and challenging aspects of veterinary work, few have explicitly explored the possibility that fulfillment and distress may arise from the same domains of veterinary practice.

Context is particularly relevant in veterinary medicine, where clinicians routinely navigate competing responsibilities to patients, clients, colleagues, employers, and the profession itself (15). Clinical decisions are often influenced by factors extending beyond medical considerations alone, including client goals, financial limitations, available resources, workplace culture, and organizational constraints (22). These realities have contributed to growing interest in Spectrum of Care (SpOC), an approach that encourages veterinarians to consider a range of evidence-informed diagnostic and treatment options and to tailor care recommendations to the circumstances of individual patients and clients while maintaining animal welfare (20, 23–29). While SpOC has gained increasing attention within veterinary education, professional organizations, and clinical practice, implementation remains variable, with concerns regarding professional expectations, communication challenges, and perceptions of acceptable standards of care identified as barriers to broader adoption (22, 30, 31). Although SpOC is frequently discussed as a strategy for improving access to care (23), it may also influence how veterinarians experience ethical complexity, professional responsibility, and career satisfaction (25, 32).

The findings reported here emerged during a broader mixed-methods study evaluating a workshop designed to support veterinarians in practicing SpOC. Although the original study focused on SpOC implementation and veterinary wellbeing, participant interviews and survey responses revealed an unexpected and recurring pattern related to career satisfaction. Rather than describing separate sources of satisfaction and dissatisfaction, participants repeatedly characterized the same domains of practice as producing both outcomes, depending on the circumstances in which they were experienced. This recurring pattern emerged across multiple interviews and was not anticipated as part of the original study objective.

The objective of this secondary qualitative analysis was therefore to explore this unanticipated finding and examine how veterinarians described the relationship between professional fulfillment and distress. Specifically, this study sought to identify the domains of veterinary practice that participants associated with both fulfillment and distress and to explore the contextual factors that appeared to influence where experiences within these domains fell along the fulfillment-distress continuum.

## 2 Methods

This manuscript reports a secondary qualitative analysis of data collected as part of a broader mixed-methods evaluation of a SpOC workshop for veterinarians. The primary study explored the impact of workshop participation on confidence in practicing SpOC and selected indicators of veterinary wellbeing. The workshop was a four-hour educational intervention designed to support veterinarians in practicing SpOC through case-based discussion, facilitated reflection, and communication exercises. The broader study employed a mixed-methods design that included questionnaires administered before, immediately after, and three months post-intervention. Additional details regarding the workshop design and evaluation have been reported elsewhere (33). The present study focuses on an emergent finding related to professional satisfaction and dissatisfaction, which was identified during qualitative analysis but was not part of the original study objectives. This secondary analysis was conducted using data collected as part of the approved study protocol and was consistent with the scope of participant consent and Research Ethics Board approval.

### 2.1 Recruitment and participants

Participants were recruited through veterinary professional networks, including direct email distribution and social media promotion. Recruitment was supported by several veterinary organizations including the Ontario Veterinary Medical Association, regional veterinary associations, and the College of Veterinarians of Ontario (CVO). Eligible participants were licensed veterinarians working primarily in clinical practice. Participation in the study was voluntary, and participants self-selected into both the workshop and research components.

Twenty-two veterinarians contributed qualitative data to the broader study through questionnaire open-text responses, and 17 of these participants participated in follow-up interviews approximately three months after workshop participation. Qualitative findings were drawn from both questionnaire responses and interview data. Respondents represented a range of career stages, community sizes, employment situations, and practice types, although most reported working primarily in companion animal practice and had more than 10 years of clinical experience. Because demographic questionnaires were completed anonymously, participant characteristics could not be linked to individual interview participants and are reported only at the broader study level.

### 2.2 Data sources

Data sources included open-text responses collected through pre-workshop, post-workshop, and three-month follow-up questionnaires, as well as semi-structured interviews conducted approximately three months following workshop participation (34). Interviews were conducted in November 2024 using Microsoft Teams (Microsoft Corporation, Redmond, United States) (35), audio-recorded, automatically transcribed, and subsequently reviewed for accuracy by the first author (SR). Questionnaires were administered using Qualtrics (Qualtrics, Provo, UT, United States) (36) and were available in English. Responses were collected anonymously between July 2024 and February 2025.

The semi-structured interviews explored participants’ experiences applying workshop concepts in practice, navigating barriers to care, and implementing SpOC approaches. Although the questions were not specifically designed to explore career satisfaction, discussions repeatedly returned to factors influencing professional fulfillment and distress, prompting the present analysis. The interview guide is provided in Supplementary Material.

### 2.3 Data analysis

Data were analyzed using reflexive thematic analysis (37, 38). Analysis was inductive and data-driven, allowing themes to emerge from participants’ accounts rather than from a predetermined theoretical framework. Coding and theme development were conducted using standard software (NVivo 14, Release 14.24.3, Denver, United States) (39).

Open-text questionnaire responses and interview transcripts were analyzed as a single qualitative dataset. Coding was conducted across both data sources concurrently, and themes were developed inductively using material from questionnaires and interviews. Questionnaire responses provided additional examples and perspectives but were not analyzed separately or used for formal triangulation. The analysis involved iterative processes of familiarization, coding, theme development, review, refinement, and interpretation. Although the original study was designed to explore the impact of a workshop on SpOC, the present analysis focused on a distinct pattern that emerged inductively from participant accounts regarding the relationship between professional fulfillment and distress. Subsequent analysis focused on exploring this pattern and identifying the contextual factors that appeared to influence whether professional experiences were perceived as fulfilling or distressing.

To support analytic rigor, the primary researcher (SR) engaged in ongoing reflexive practice throughout data collection and analysis through memoing and critical self-reflection (40). Two co-authors (LVP, AJ) independently coded two interview transcripts each and subsequently participated in separate debriefing discussions with the primary researcher to compare interpretations and discuss coding decisions. Participants were given the opportunity to review their interview transcripts and provide clarifications or feedback.

## 3 Results

Twenty-two veterinarians contributed qualitative data through questionnaire open-text responses, and 17 participated in follow-up interviews three months after workshop completion. Data sufficiency was achieved before all interviews were coded, as additional interviews did not yield substantively new insights for theme development (41).

### 3.1 The duality of veterinary career satisfaction

An unexpected finding was that participants did not describe distinct sources of career satisfaction and dissatisfaction. Instead, the same domains of veterinary practice were frequently discussed as both fulfilling and distressing depending on the circumstances in which they were experienced. Several participants explicitly described veterinary practice in dual terms. As one veterinarian stated, *“there’s lots to love … practice is also challenging”* (P18). Five domains illustrated this duality: client relationships, professional culture, intellectual challenge, clinical outcomes, and ethical complexity (Figure 1). Each domain was described as functioning as either a source of fulfillment or a source of distress, depending on context. Across all domains, contextual factors appeared to influence whether experiences were perceived as rewarding or burdensome.

**Figure 1:**
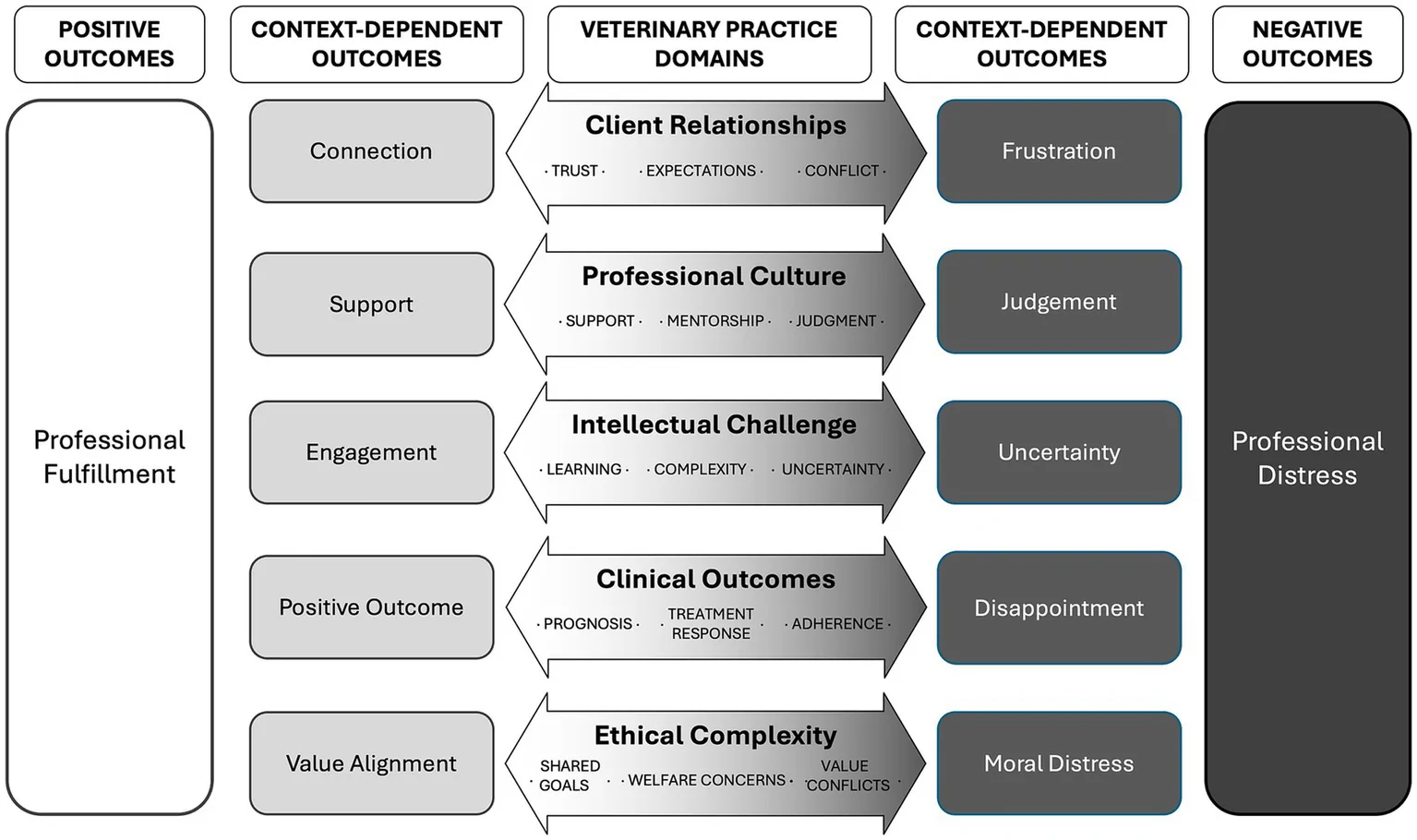
The duality of veterinary career satisfaction. Participants described five domains of veterinary practice that could contribute to either fulfillment or distress. Contextual factors, including workplace culture, professional autonomy, value alignment, organizational constraints, and fear of judgement, appeared to influence how these domains were experienced.

### 3.2 Client relationships: connection and frustration

Relationships with clients and their animals were among the most frequently cited sources of professional satisfaction. Participants described meaningful interactions as energizing and central to their sense of purpose.

*“I think what really fuels me a lot is client interactions.”* (P15)

*“You get some really fun clients where you are like, ‘I have to book more time for you or I’m gonna run super late’ because you just enjoy chatting and enjoy hanging out with their pets.”* (P1)

Some participants explicitly described both rewarding and frustrating aspects of client relationships. As one veterinarian reflected:

*“I like just getting to know my clients … I was surprised how much time I spent just sort of trying to please my client versus doing medicine.”* (P3)

At the same time, these relationships could be emotionally exhausting when participants felt their efforts were unsuccessful, unrecognized, or dismissed.

*“I’m surprised at how combative people can be about information that’s coming from me versus the kid at the pet store.”* (P1)

*“When you have done an hour conversation with someone and they walk out, and they do not do any of the things you recommended, it impacts every person on the team.”* (P8)

Others described frustration arising when broader priorities appeared to reduce the value placed on veterinary care:

“*During the pandemic, owners willing[ly] spent money on their pet’s care. There was the least resistance or questioning of quotes that [I have] ever experienced. However, since pandemic travel restrictions have been removed, pet care needs are less important compared to vacations.”* (Questionnaire response)

These accounts illustrate how client relationships could function as both a source of satisfaction and a source of frustration, depending on how interactions unfolded.

### 3.3 Professional culture: belonging and disillusionment

Participants described professional culture in paradoxical terms. Supportive clinic teams were consistently associated with wellbeing, job satisfaction, and resilience.

*“Recently I’m surprised by what an enormous difference your team around you makes … in the quality of … your work life … and your life in general.”* (P13)

In contrast, broader professional norms were often characterized as judgmental, discouraging, or misaligned with participants’ values.

*“The way the profession has been turning… COVID really brought out the worst and the best, and opportunities and opportunistic behaviour… those things are surprising, but not surprising. They disappoint more than surprise.”* (P18)

One participant described discomfort with what they perceived as a longstanding tendency within the profession to judge clients:

*“I think in the past in veterinary medicine, we have always tended to blame the client … it has always made me uncomfortable.”* (P13)

While local workplace culture often functioned as a buffer against stress, broader professional culture was frequently experienced as a source of dissatisfaction and moral discomfort.

### 3.4 Intellectual challenge: engagement and uncertainty

Many participants described the intellectual challenge of veterinary medicine as a key source of professional fulfillment. Solving complex cases, continuous learning, and encountering novel situations contributed to professional enjoyment and engagement.

*“For years, you know, people wanted to hear that you love the variety and the challenge—and I hated that… Where I’m really enjoying practice, I am enjoying the challenge, I am enjoying doing new things, whereas new things used to terrify me. So, so the things that I said I liked for a long time but did not like, I actually like now.”* (P15)

*“I love that I still see new different things. I love medicine. I love practicing medicine. Like I love the puzzle of figuring out what’s wrong. Pulling together so many different facts and so I do, I love medicine.”* (P13)

One participant described the uncertainty inherent to emergency medicine as both a source of enjoyment and emotional exhaustion:

*“I still love and have always loved the mystery … I’m surprised at how emotionally tired I am.”* (P2)

However, many participants also described the cognitive demands of practice as overwhelming, particularly given rapid growth in veterinary knowledge and persistent uncertainty surrounding clinical decisions.

*“And there’s, you know, the uncomfortableness or the anxiety about what if … we did not do everything that we could have and we missed something and it kind of comes back to haunt me.”* (P2)

*“Over the years, just the volume of information that is new and that we are, you know, supposed to and expected to keep up with, right. It’s tough, definitely.”* (P16)

Participants therefore described intellectual challenge as both a source of engagement and a source of uncertainty.

### 3.5 Clinical outcomes: fulfillment and disappointment

Positive clinical outcomes were described as important sources of professional satisfaction. Participants derived substantial fulfillment from successful treatment, patient recovery, and improving animal welfare.

*“I love seeing a patient … improve and do better … or go home from the hospital because you have made them better.”* (P13)

In contrast, unresolved cases, unsuccessful interventions, and persistent uncertainty were described as emotionally draining and professionally frustrating.

*“Sometimes I find, like, the most frustrating outcomes are the ones that get the most testing and do not get an answer.”* (P9)

These accounts demonstrate how clinical outcomes were experienced as both rewarding and frustrating aspects of clinical practice.

### 3.6 Ethical complexity: value alignment and moral distress

Ethical complexity represented the clearest example of duality within veterinary practice. Participants described ethical decision-making as emotionally taxing when patient needs, client circumstances, business realities, and professional ideals appeared misaligned.

*“I have to provide care for an animal, but I also have to ensure that that’s not compromising the livelihood and the care of the team that I work with.”* (P18)

Several participants described distress when financial limitations, clinic policies, or other external constraints prevented them from providing care consistent with their values. Ethical conflict was therefore often linked not only to individual cases, but also to broader organizational and systemic structures.

*“It feels like being a middleman… You’re between being a helper for the client, but also making sure you are adhering to your responsibilities as an employee.”* (P12)

*“I have colleagues… who work for corporate where, you know, ‘no blood, no drug.’ And that’s very hard for them.”* (P5)

One respondent emphasized the distinction between burnout and moral injury:

*“It is important to differentiate between burnout and moral injury. Although burnout is certainly present in vet med, it’s the moral injury that makes vet med intolerable.”* (Questionnaire response)

At the same time, many participants described practicing SpOC as a mechanism for navigating these tensions. Contextualized approaches enabled them to provide care that aligned more closely with both their professional values and clients’ realities, providing a powerful source of fulfillment for the whole team.

*“You just see the morale jump because nobody wants to let that pet go. They just want to provide the care. And so, I think doing that, or at least working with spectrum of care when we can, is really good for the morale of the team because we are all in this for the same reason.”* (P3)

These accounts suggest that ethical complexity can function as either a source of distress or a source of fulfillment depending on whether veterinarians feel able to provide care that aligns with both their professional values and the realities facing clients and veterinary teams.

## 4 Discussion

The most notable finding from this study was not the identification of factors associated with veterinary career satisfaction, but the observation that participants frequently described the same domains of practice as sources of both fulfillment and distress. A consistent pattern across interviews was that the domains themselves were not inherently positive or negative. Instead, participants described the quality of their experiences within these domains as shaping whether they were perceived as rewarding or burdensome. Meaningful client relationships could be energizing, yet difficult interactions could be emotionally exhausting. Intellectual challenge could foster engagement and professional growth, while also creating anxiety and self-doubt. Similarly, ethical decision-making could generate either professional fulfillment or moral distress depending on the circumstances. This finding aligns with previous descriptions of the “paradox of compassionate work,” in which the same caring relationships that foster compassion satisfaction can also contribute to compassion fatigue and emotional exhaustion (2, 8). However, participants in this study described a similar duality extending beyond client and patient relationships to encompass professional culture, clinical reasoning, clinical outcomes, and ethical decision-making, suggesting that this paradox may be a more pervasive feature of veterinary practice than previously recognized.

The finding also expands upon existing models of meaning and wellbeing in veterinary medicine. Cake et al. identified relationships, challenge, competence, and contribution as important sources of meaning and professional fulfillment (42), while other studies have highlighted client interactions, intellectual stimulation, and helping animals as important contributors to career satisfaction (2, 4, 18, 42–44). However, participants in the present study described these same domains as potential sources of frustration, disappointment, and distress when contextual factors limited their ability to achieve desired outcomes or act in accordance with their values. This suggests that domains traditionally framed as contributors to wellbeing may not be inherently protective but instead exist along a continuum where their impact is shaped by circumstance.

### 4.1 Context as the determinant of satisfaction and distress

The duality observed across all five domains suggests that veterinary career satisfaction may be shaped less by the presence of particular professional experiences than by the context in which those experiences occur. Participants did not describe client relationships, clinical complexity, professional culture, or ethical decision-making as inherently rewarding or inherently burdensome. Rather, the impact of these experiences appeared to depend on whether veterinarians felt supported, empowered, and able to align their actions with their professional values. This interpretation is consistent with broader wellbeing literature demonstrating that workplace factors such as autonomy, social support, organizational culture, and value alignment strongly influence professional engagement, burnout, and job satisfaction (12, 13, 45). Studies of veterinary teams have similarly found that workplace culture and team effectiveness are closely associated with both wellbeing and career satisfaction (4, 45, 46).

Across interviews, participants repeatedly described supportive teams as transforming challenging situations into manageable and meaningful experiences. Team members who shared responsibility, communicated openly, and approached problems collaboratively appeared to buffer the emotional burden associated with difficult clients, complex cases, and uncertain outcomes. These findings align with previous research demonstrating that healthy team environments promote resilience, engagement, and retention, whereas toxic workplace cultures contribute to burnout, disengagement, and emotional exhaustion (47–49).

Professional autonomy emerged as another important contextual influence. Participants frequently described frustration when they felt constrained by organizational policies, corporate structures, financial realities, or perceived regulatory expectations. Similar findings have been reported elsewhere, where reduced autonomy and inability to act according to professional judgement have been linked to moral distress and decreased job satisfaction (15–17, 45, 50). In contrast, participants who felt empowered to exercise professional discretion and tailor recommendations to the circumstances of individual clients often described greater satisfaction and confidence in their decision-making. These findings suggest that autonomy may act as a moderator between the demands of practice and their impact on wellbeing.

Alignment between professional values and achievable actions also appeared central to participants’ experiences. Consistent with literature on moral distress, dissatisfaction was often greatest when veterinarians felt unable to provide care they believed was ethically appropriate due to external constraints. However, participants reported much lower levels of distress when they felt they had identified a care plan that respected both their professional responsibilities and the realities facing the client. This observation supports existing literature on moral distress, moral injury, and professional identity, which suggests that wellbeing is influenced not only by clinical outcomes but also by the extent to which veterinarians feel able to act in accordance with their values and professional conception of what constitutes good veterinary care (15–17, 19, 51).

Taken together, these findings suggest that the domains identified in this study are best understood as relatively stable features of veterinary practice, whereas satisfaction and dissatisfaction reflect context-dependent responses to those domains. Client relationships, intellectual challenge, clinical responsibility, and ethical complexity are unlikely to be removed from veterinary medicine; indeed, many participants cited these same features as reasons they entered and remained in the profession. The findings therefore imply that efforts to improve veterinary wellbeing may be most effective when focused not on eliminating difficult aspects of practice, but on creating conditions that allow veterinarians to experience those aspects as meaningful, manageable, and aligned with their professional values. Such an interpretation provides a useful framework for understanding how the same professional experience can become either a source of fulfillment or a source of distress.

### 4.2 Spectrum of care as a mechanism for reducing distress and enhancing satisfaction

Participants’ accounts suggest that SpOC may influence wellbeing not by eliminating the challenges inherent to veterinary practice, but by changing how veterinarians experience them. Across interviews, ethical complexity emerged as both one of the greatest sources of distress and one of the domains most amenable to positive change through contextualized care. Participants frequently described frustration when external constraints prevented them from pursuing what they believed was the most appropriate course of action. These findings align with literature suggesting that ethical distress may be influenced not only by the presence of challenging circumstances, but also by veterinarians’ ability to navigate those circumstances in ways that align with their professional values (7). Participants frequently described greater satisfaction when they were able to identify care plans that balanced patient needs, client circumstances, and professional obligations.

Several participants described SpOC as providing a framework that legitimized flexible decision-making and expanded the range of options available to both the clinic team and clients. Rather than viewing alternative approaches as inferior versions of care, participants who embraced a contextualized approach described greater comfort discussing a range of diagnostic and treatment strategies. This observation is consistent with the broader SpOC literature, which emphasizes that quality veterinary care can be delivered through multiple evidence-informed pathways rather than a single idealized approach (20, 23–29).

Importantly, the benefits described by participants extended beyond patient care and access to veterinary services. Several veterinarians reported that contextualized care reduced ethical tension, improved team morale, strengthened client relationships, and increased confidence when navigating difficult conversations. These findings align with growing literature suggesting that SpOC and contextualized care may offer benefits beyond expanding treatment options alone. Skipper et al. argued that contextualized care should be viewed as a foundational clinical approach rather than simply a response to financial limitations, while Brown et al. emphasized that SpOC encompasses broader considerations of communication, clinical reasoning, and ethical decision-making (24, 32, 52). Participants in the present study similarly described SpOC as a framework that helped align clinical decision-making with both professional values and real-world constraints. Given that moral distress commonly arises when veterinarians perceive themselves as unable to provide care consistent with their ethical obligations or professional standards (15–17), SpOC may represent one mechanism through which this tension can be reduced by creating clinically and ethically acceptable pathways that remain achievable within contextual limitations (53).

Participants often discussed SpOC in terms that extended beyond clinical decision-making. While SpOC is frequently presented as a strategy for improving access to veterinary care (23, 27, 28), participants also described it as a means of reconciling competing responsibilities to patients, clients, colleagues, employers, and themselves. This finding resonates with literature describing veterinary medicine as a profession characterized by competing obligations and ethical complexity (19–21). Ethical conflict frequently arises from balancing patient welfare, client circumstances, business sustainability, and professional obligations (19, 20), and these competing pressures are recognized contributors to moral distress (16, 17). Rather than removing these tensions, participants described SpOC as helping them navigate them more effectively. Viewed through this lens, SpOC may function not only as a clinical framework but also as a professional coping and sense-making framework that helps veterinarians navigate the inherent complexity of practice while maintaining alignment with their professional identity (51).

At the same time, participants’ experiences suggest that the benefits of SpOC are dependent on the broader professional environment. When veterinarians feared judgement from colleagues, criticism from regulatory bodies, or accusations of providing substandard care, they reported hesitancy to fully embrace contextualized approaches. Similar concerns have been described elsewhere in the veterinary literature. Research examining SpOC implementation has identified professional expectations surrounding *gold-standard* care as a significant barrier to adopting more flexible approaches (22, 24, 25), while studies of the hidden curriculum suggest that informal professional norms may reinforce perceptions that deviation from idealized care represents clinical compromise or professional failure (54, 55). These concerns mirror broader findings that fear of complaints, peer judgement, and professional scrutiny can influence clinical decision-making and contribute to defensive practice behaviours (14, 56–59). Collectively, these influences may limit the ability of veterinarians to fully realize the benefits of contextualized care, even when they personally support its principles.

Taken together, these findings suggest that SpOC may represent more than an access-to-care initiative. By supporting veterinarians in developing care plans that are both medically appropriate and contextually achievable, it may help restore alignment between professional values and clinical action. This interpretation is consistent with literature linking value congruence, professional autonomy, and ethical agency to improved wellbeing and reduced moral distress (15–17, 51). Participants repeatedly described value alignment as an important determinant of whether professional experiences were perceived as fulfilling or distressing. Consequently, the potential impact of SpOC may extend beyond access to care and patient outcomes to include practitioner wellbeing. Future research should explore whether increased confidence in practicing contextualized care is associated with measurable reductions in moral distress, burnout, or compassion fatigue and increased levels of professional satisfaction.

### 4.3 Professional culture as a moderator of veterinary wellbeing

Findings from this study suggest that professional culture may play an important role in determining whether common domains of veterinary practice are experienced as sources of fulfillment or distress. Although participants frequently described supportive clinic teams as major contributors to job satisfaction, they often characterized the broader profession as judgmental, perfectionistic, and resistant to flexibility. These perceptions are consistent with studies describing the influence of the hidden curriculum in veterinary medicine, whereby informal norms and expectations shape professional identity and behaviour beyond the formal curriculum (54, 55). Within such environments, veterinarians may internalize narrow definitions of success and failure that influence how they interpret everyday clinical experiences.

Participants’ descriptions of fear were particularly notable. Fear of peer judgement, regulatory scrutiny, client complaints, and making mistakes emerged across discussions of client communication, clinical decision-making, and implementation of contextualized care. Similar concerns have been documented in studies examining defensive veterinary practice and the impact of complaints on professional wellbeing (14, 56–58). Importantly, participants’ accounts suggest that fear may function not only as a barrier to SpOC but also as a broader determinant of professional satisfaction by influencing how veterinarians experience uncertainty, responsibility, and clinical decision-making.

Perfectionistic expectations may further contribute to this process. Perfectionism is well recognized within veterinary medicine and has been associated with increased stress, burnout, and moral distress (16, 50). Participants frequently described an implicit expectation that *good* veterinarians should always identify the correct diagnosis, recommend the best treatment, and achieve positive outcomes. When these expectations were unattainable, as is often the case in real-world practice, the resulting gap between ideals and reality appeared to contribute to frustration and dissatisfaction. Conversely, participants who adopted more flexible, contextualized approaches often described greater acceptance of uncertainty and greater satisfaction with achievable outcomes.

Viewed through the lens of the duality model proposed in this study, professional culture may influence where veterinarians fall along a continuum between fulfillment and distress. A culture that normalizes uncertainty, supports collaborative decision-making, and legitimizes multiple acceptable approaches to care may shift common professional experiences toward satisfaction. In contrast, cultures that emphasize perfection, judgment, and adherence to idealized standards may increase the likelihood that those same experiences are perceived as distressing. Professional culture, therefore, appears not merely to be another domain influencing wellbeing, but a contextual force shaping how all other domains are experienced.

### 4.4 Implications for veterinary wellbeing and future interventions

These findings support growing recognition that veterinary wellbeing cannot be understood solely through the lens of individual resilience or coping capacity. Although self-care strategies, mindfulness, and stress-management approaches are commonly promoted within the profession (7), there is growing recognition that workplace culture, team functioning, professional norms, and other systemic influences also shape wellbeing (60, 61). Participants in the present study similarly described professional culture, team dynamics, autonomy, value alignment, collaborative communication, and access to flexible care pathways as important influences on whether common aspects of practice were experienced as fulfilling or distressing. Together, these findings suggest that interventions designed to improve wellbeing should focus not only on supporting individual veterinarians but also on creating workplace environments that enable value-aligned and context-appropriate practice.

The proposed duality model also has implications for veterinary education. Client interactions, ethical decision-making, clinical responsibility, and professional complexity cannot be removed from veterinary practice. Traditional approaches to wellbeing often emphasize resilience, self-care, and coping strategies, whereas participants frequently described challenges arising from systemic and cultural influences beyond their individual control. Efforts to improve wellbeing may therefore be most effective when focused on modifying the conditions under which these domains are experienced. Supportive team cultures, increased autonomy, validation of contextualized care, and training that normalizes uncertainty may shift common professional experiences from sources of distress toward sources of satisfaction (45, 48, 62–65). Educational initiatives that normalize uncertainty, encourage reflective practice, explore multiple acceptable approaches to care, and support development of professional identity may help prepare veterinarians to navigate complexity without interpreting every deviation from idealized care as failure (22, 28, 51, 52, 55, 66).

Importantly, the findings suggest that interventions aimed at increasing access to care and improving practitioner wellbeing may not be independent goals. Consistent with the literature, participants frequently described contextualized care and SpOC approaches as benefiting both clients and veterinary professionals by reducing ethical tension and supporting value-aligned practice (32, 52, 67). This raises the possibility that initiatives designed to expand flexible care options may simultaneously improve practitioner wellbeing, an area warranting further investigation.

Future research should examine whether the duality model identified in this study is observable in larger and more diverse veterinary populations, including different practice types and geographic regions. Quantitative studies could explore whether factors such as professional autonomy, workplace culture, and value alignment function as mediators between common domains of practice and wellbeing outcomes. Longitudinal research may also help clarify how veterinarians move between experiences of fulfillment and distress over time and identify factors that facilitate these transitions.

## 5 Limitations

Several limitations should be considered when interpreting these findings. Participants were recruited from a workshop focused on SpOC and therefore represented a self-selected group of veterinarians with an existing interest in contextualized care. Their views may differ from those of veterinarians who are less familiar with, or less supportive of, SpOC principles. Although this may limit transferability, it also provided an opportunity to explore the experiences of clinicians actively attempting to navigate the challenges associated with providing accessible and context-appropriate care.

As a qualitative study, the objective was to generate an in-depth understanding of participants’ experiences rather than to estimate the prevalence of specific perspectives or experiences within the broader veterinary profession. The findings therefore should not be interpreted as evidence that all veterinarians experience satisfaction and dissatisfaction in the manner described by participants in this study. Future research involving larger and more diverse populations will be necessary to determine the extent to which the proposed duality model is applicable across practice types, career stages, and geographic regions.

The themes reported in this manuscript emerged as unanticipated findings from a broader study evaluating a SpOC workshop. Consequently, interview guides and questionnaire instruments were not specifically designed to explore career satisfaction. While this limits the depth to which some concepts could be examined, it also strengthens confidence that the identified themes reflected issues that participants considered salient enough to raise spontaneously rather than topics introduced directly by the research team.

Because data were collected following participation in a workshop focused on SpOC, it is possible that workshop content influenced how participants reflected on and described their professional experiences. Discussions of flexibility, contextualized care, professional values, and access to care may have sensitized participants to these issues. Consequently, participant accounts may not fully reflect perspectives that would have emerged in the absence of the workshop. However, the themes reported in this manuscript emerged spontaneously across interviews and questionnaire responses rather than from questions specifically focused on career satisfaction, suggesting that participants considered these issues salient to their professional experiences.

Finally, as with all qualitative research, findings were co-constructed through the interaction between participants and the researcher. As a practicing veterinarian and workshop facilitator, the lead researcher brought both subject-matter expertise and prior assumptions regarding SpOC and veterinary wellbeing to the study. Reflexive practices, including memoing and critical self-reflection, were used throughout the study to examine how these perspectives might influence interpretation and to enhance the trustworthiness of the findings. In addition, two co-authors independently reviewed a subset of transcripts and participated in debriefing discussions with the lead researcher. Nevertheless, researcher perspectives inevitably influenced the interpretation of findings.

## 6 Conclusion

An unexpected finding from this study was that veterinarians did not describe separate sources of career satisfaction and dissatisfaction. Instead, client relationships, professional culture, intellectual challenge, clinical outcomes, and ethical complexity functioned as sources of either fulfillment or distress depending on context. These findings suggest that veterinary wellbeing may be better understood as a dynamic, context-dependent phenomenon arising from shared domains of professional practice rather than distinct categories of positive and negative workplace factors.

Participants described workplace culture, professional autonomy, value alignment, and access to alternative care pathways as important influences on whether common professional experiences were perceived as fulfilling or distressing. SpOC emerged as one potential mechanism for supporting value-aligned practice by helping veterinarians navigate competing obligations while maintaining ethical integrity and responsiveness to client circumstances. At the same time, fear of judgment, professional norms, and elements of the hidden curriculum were identified as barriers that can limit the adoption of flexible approaches to care.

Collectively, these findings suggest that improving veterinary wellbeing may depend less on eliminating difficult aspects of veterinary work and more on creating conditions that allow those experiences to be interpreted as meaningful, manageable, and professionally rewarding. Efforts to foster supportive professional cultures, normalize contextualized care, and create environments in which veterinarians can practice in accordance with their values may help shift common professional experiences from sources of distress toward sources of fulfillment. Further research is needed to evaluate the proposed duality model and its implications for veterinary wellbeing and access to care.

## Data Availability

Participant consent for data sharing was not obtained, and the qualitative data contain potentially identifiable information. As a result, the dataset cannot be publicly shared or made available upon request. Further inquiries can be directed to the corresponding author/s.
