# Supplementary material for: The duality of veterinary career satisfaction: shared domains of fulfillment and distress in veterinary practice
Source: Front Vet Sci. 2026 Oct 8;13:1958614. doi: 10.3389/fvets.2026.1958614 (PMC13688873; doi:10.3389/fvets.2026.1958614)
Supplement: Supplementary file 1 [file Supplementary_file_1.docx]

Semi-structured interview guide

**Ice-breaker questions:**

- How long have you been in practice?
- What do you love about practice?
- What has surprised you about practice?

**In your opinion, who are the stakeholders involved in addressing client barriers to accessing veterinary care and what are their roles/responsibilities?**

- What role do you play in the face of client barriers to accessing veterinary care?
- What challenges do you face when dealing with barriers to accessing veterinary care?

**What (if any) changes have you made or actions have you taken in terms of Spectrum of Care since participating in the workshop?**

- What has been the impact of these implemented changes?
- In the past 3 months, how many cases visits have you used a spectrum of care approach with?
- How do you approach sharing the spectrum of options with clients?
- Thinking about the range of options presented to the client, where along the range was selected (low/middle/high)?
- What were the patient outcomes?
- What aspects of the SoC approach taken in these case(s) were viewed positively by the veterinary team?
- What aspects of the SoC approach taken in these case(s) were viewed negatively by the veterinary team?

**What (if any) additional training and/or resources would help enable you to increase the spectrum of care you practice?**
